# A genetic epidemiological model to describe resistance to an endemic bacterial disease in livestock: application to footrot in sheep

**DOI:** 10.1186/1297-9686-41-19

**Published:** 2009-01-26

**Authors:** Gert Jan Nieuwhof, Joanne Conington, Stephen C Bishop

**Affiliations:** 1 Meat and Livestock Commission, Milton Keynes MK6 1AX, UK; 2 ADHIS, DPI Bundoora, Victoria, Australia; 3 Sustainable Livestock Systems Group, SAC, West Mains Road, Edinburgh EH9 3JG, UK; 4 The Roslin Institute and Royal (Dick) School of Veterinary Studies, University of Edinburgh, Roslin, Midlothian EH25 9PS, UK

## Abstract

Selection for resistance to an infectious disease not only improves resistance of animals, but also has the potential to reduce the pathogen challenge to contemporaries, especially when the population under selection is the main reservoir of pathogens. A model was developed to describe the epidemiological cycle that animals in affected populations typically go through; viz. susceptible, latently infected, diseased and infectious, recovered and reverting back to susceptible through loss of immunity, and the rates at which animals move from one state to the next, along with effects on the pathogen population. The equilibrium prevalence was estimated as a function of these rates. The likely response to selection for increased resistance was predicted using a quantitative genetic threshold model and also by using epidemiological models with and without reduced pathogen burden. Models were standardised to achieve the same genetic response to one round of selection. The model was then applied to footrot in sheep. The only epidemiological parameters with major impacts for prediction of genetic progress were the rate at which animals recover from infection and the notional reproductive rate of the pathogen. There are few published estimates for these parameters, but plausible values for the rate of recovery would result in a response to selection, in terms of changes in the observed prevalence, double that predicted by purely genetic models in the medium term (*e.g*. 2–5 generations).

## Introduction

Preventive measures and lost production due to endemic disease form an important component of the costs of production in many livestock production systems [1], and they also affect animal welfare and marketability of breeding stock. It is well known that, for many diseases, resistance has a genetic component [2] and selection for disease resistance has long been considered a promising way to reduce disease prevalence *e.g*. [3].

Selection for resistance to an infectious disease has the added benefit that it may reduce the pathogen burden, especially when the population under selection is the main reservoir of pathogens. This will lead to an additional reduction in prevalence, in addition to the direct genetic effect, as a result of reduced contamination from infectious animals, *e.g*. [4].

The phenotype used in selection for disease resistance is often a score, which includes a class of healthy animals, and one or more classes of affected animals. In the case of endemic diseases the vast majority of animals at any one time may be classified as healthy, and this limits the opportunity for intense selection. In a threshold model, that is appropriate for this type of data, prevalences that are much lower than 50% also lead to low heritabilities on the observed scale. A successful selection programme can therefore be expected to decrease the subsequent response to selection through increased resistance and decreased pathogen burden.

Anderson and May [5] describe the spread of a microparasitic (viral or bacterial) infection through a population of animals using a so-called SIR model, based on the rates at which susceptible (*S*) animals are infected (*I*) and then recover or are removed (*R*). A key parameter is *R*_0_, which is the number of secondary infections caused by the first infected animal. One or more of these rates can be under genetic control and hence affect *R*_0_. This model can be extended in various ways; for instance Bishop and MacKenzie [6] have described how a disease that is spread from animal to animal may or may not lead to an epidemic, and Nath *et al*. [7] have explored the consequences of selection to alter different model parameters. To make these models more applicable to typical livestock bacterial infections, Bishop *et al*. [8] have considered a disease in which the pathogen survives for some time in the environment (*E*) from where it can infect susceptible animals. This was termed a SEIR model.

In the above models, the assumption is that recovered animals are no longer susceptible to the disease, and in a closed population without re-infection these models will therefore always lead to zero prevalence, either because there are no susceptible animals left or because the disease has died out. This outcome is inappropriate for typical endemic diseases, which often have a more or less stable prevalence over time. This stable prevalence, the endemic equilibrium, may be due to recovered animals losing their resistance and becoming susceptible again, or it may simply be a consequence of a continued introduction of new susceptible animals, *e.g*. offspring.

Building on the SEIR model [8], we introduce two new aspects to the model: (i) a period of latency (*L*) in which animals are infected but not yet infectious and (ii) loss of immunity so that recovered (*R*) animals can revert back to susceptible. This creates a SELIRS model. Further, we consider the SELDCRS model in which animals can be diseased and infectious (*D*) or a carrier, which is infectious but no longer clinically diseased (*C*). This model can be used to distinguish direct effects related to the number of diseased animals from indirect effects related to pathogen burden, by manipulating relative rates associated with *D *and *C*.

Footrot is an infectious disease of sheep caused by bacteria (*Dichelobacter nodosus*) that survive in soil for a limited time. The prevalence in adult sheep in Britain is around 6% [9]. Resistance to footrot has been shown to be heritable [10-12], and selection for increased resistance is feasible.

The aim of this study was to develop SELIRS and SELDCRS epidemic models, in which pathogens survive in the environment for a limited time. The models were applied to footrot in sheep and used to predict the changes in prevalence of footrot over time if selection is for resistance to the disease, accounting for the disease dynamics. The predicted progress in terms of reduction in prevalence was compared with a model that ignores epidemiological effects.

## Methods

### Definition of epidemic models

#### Case 1: Infectious and diseased animals are equivalent

An overview of all symbols and abbreviations used and their definitions is given in Table 1. Consider a population of *N *individuals which, at time *j*, consists of *S *susceptible, *L *latently infected, *I *infected and *R *recovered animals. It is assumed that it is only category *I *animals that show clinical signs of disease and are infectious. Environmental contamination is quantified by the concept of an infectious dose; therefore at time *j *there are *E *infectious doses of the pathogen in the environment. Following Bishop *et al*., [8], and approximating the discrete process (*e.g*. daily steps) by a continuous one, the SELIRS model is defined by the following five differential equations:

**Table 1:** Summary and definition of symbols used in epidemiological models

Symbol	Definition
S	The number of susceptible animals
*E*	The number of infectious doses in the environment
*L*	The number of latently infected animals
*I*	The number of diseased and infectious animals in SELIRS model
*D*	The number of diseased and infectious animals in SELDCRS model
*C*	The number of infectious carriers in the SELDCRS model
*N*	The total number of host animals in the population
*R*	The number of recovered animals
*ν*	The rate at which latently infected animals develop clinical signs and become infectious
*μ*	The rate at which infectious doses (bacteria) die in the environment, other than by host animals
*ω*	The rate at which infectious doses (bacteria) are physically removed by each host animal in the population
*χ*	The total rate at which infectious doses (bacteria) are removed from the environment, calculated as *μ *+ *ω*N
*γ*	The rate at which diseased and infectious animals stop showing clinical signs and are no longer infectious, in the SELIRS model
*κ*	The rate at which diseased animals stop showing clinical signs, while continuing to be infectious in the SELDCRS model
*α*	The rate at which infectious animals that no longer show clinical signs of the disease stop being infectious in the SELDCRS model
*λ*	The rate at which recovered animals lose resistance and become susceptible
*ξ*	The rate at which susceptible animals become infected by 1 unit of infectious dose in the environment
*φ*	The rate at which an infectious animal sheds infectious doses in the environment
*p*	The prevalence of the disease as observed from clinical signs
*R*'	The notional reproductive rate of the infectious disease

(1) *dS/dt *= -*ξES*+*λR*.

(2) *dE*/*dt *= *φI*-*μE*-*ωNE*.

(3) *dL/dt *= *ξES*-*νL*.

(4) *dI/dt *= *νL*-*γI*.

(5) *dR/dt *= *γI*-*λR*.

where *ξ *is the rate at which susceptible animals become infected (latent) per infectious dose, *λ *is the rate of loss of immunity, *ν *is the rate at which latent animals develop clinical signs and become infectious, *φ *is the rate at which infectious doses are shed by infected animals, *μ *is the rate at which infectious doses die (other than by host animals), *ω *is the rate at which a host animal physically removes infectious doses (*e.g*. by ingestion, adherence to the animal or squashing them) and *γ *is the rate at which infectious animals become immune. All rates are non-negative.

Two properties of this model are of importance, the notional reproductive rate (*R'*) and the equilibrium prevalence. In standard epidemiological models the basic reproductive ratio, *R*_0_, is the number of infections caused by a single infected animal in a wholly susceptible population, during the course of its infectious period. The equivalent in the SELIRS model is the number of secondary infections due to a single infectious animal, for the time period over which the infectious material remains in the environment. Bishop *et al*. [8] have derived an expression for the notional reproductive rate in a SEIR model as:

(6) $$R'=\frac{\phi \xi N}{\chi \gamma }$$

where *χ *= *μ *+ *ωN*, *i.e*. the total rate at which the infection is removed from the environment. The same expression may be used as an approximation to *R' *in an SELIRS model (see Appendix 1), however it is not exact as the loss of immunity potentially increases the number of secondary infections in situations where the environmental contamination is long-lived and the period of immunity is short.

An equilibrium state will be reached when the number of animals in each state is the same from one day to the next. In other words, the rates of change for the numbers of each category of animal (equations 1, 3, 4, 5) are all zero, *i.e*. d*S*/d*t *= d*L*/d*t *= d*I*/d*t *= d*R*/d*t *= 0. It can be shown (see Appendix 1) that the corresponding equilibrium number of diseased and infectious animals (I*) is:

(7) $$I*=\frac{\lambda \upsilon (N-\frac{\gamma \chi }{\xi \phi })}{\upsilon \lambda +\lambda \gamma +\upsilon \gamma }.$$

Combined with (6), it follows that the equilibrium prevalence (p*), i.e. I*/*N*, is

(8) $$p*=\frac{\lambda \upsilon (1-\frac{1}{R'})}{\upsilon \lambda +\lambda \gamma +\upsilon \gamma }=\frac{\lambda \upsilon (1-\frac{1}{R'})}{\upsilon \lambda \frac{1}{\gamma }+\lambda +\upsilon }\;\frac{1}{\gamma }.$$

Hence, after rearrangement, *R' *may be defined as a function of the equilibrium prevalence as follows:

$$R'=\frac{1}{1-(1+\gamma /\upsilon +\gamma /\lambda )p*}.$$

Note that in equation (7) *φ *(infection shedding rate from animal) and *ξ *(infection rate) occur as the product *ξφ *indicating that the rate of infection depends jointly on the number of infective units spread by infected animals and the number of units required for an animal to become infected. This means that there is no need for an exact definition of the infective dose.

#### Case 2: Infectious animals with and without clinical signs

In many instances animals may be infectious, even when no clinical signs of disease are apparent, a phenomenon in some circumstances referred to as 'carrier status'. Defining clinical disease and infectious status as two separate but overlapping categories also allows greater flexibility in the exploration of the disease dynamics. This is achieved in the SELDCRS model, in which animals can be diseased and infectious (*D*) or infectious carriers who no longer show clinical signs (*C*), with *N *= *S+L+D+C+R*. Equations (1) and (3) describing change in *S *and *L *remain the same, with the remaining equations being:

(9) *dE/dt *= *φ*(*D*+*C*)-*χE*.

(10) *dD/dt *= *νL*-*κD*.

(11) *dC/dt *= *κD*-*αC*.

(12) *dR/dt *= *αC*-*λR*.

Where the new symbols are: *κ *is the rate at which diseased animals no longer show clinical signs and move to the carrier state, and *α *is the rate at which carrier state animals cease to be infectious. The total time an animal is infectious is 1/*κ*+1/*α*, rather than simply 1/*γ *as in the SELIRS model. This accounts for the supposition that infected animals may stop showing clinical signs yet continue to be infectious, rather than assuming that only animals with clinical signs are infectious. For the SELIRS and SELDCRS models to be equivalent in terms of transmission of infection then the total infectious period must be the same in both models, *i.e*. 1/*γ *= (1/*κ*+1/*α*). Note that this is proposed solely as a theoretical tool to distinguish between the effects of recovery on the animal it self (clinical signs) and on the population (infectious), which are confounded in the SELDIRS model. It is not proposed as a selection strategy. Table 2 shows a contrast of similar parameters in the SELIRS and SELDCRS models.

**Table 2:** Contrast of similar parameters in the SELIRS and SELDCRS models

Model	Symbol	Definition
(1) The number of diseased and infectious animals		
SELIRS	*I*	From state *I*, animals recover and move to state *R *(recovered) at rate *γ*, and hence are no longer infectious
SELDCRS	*D*	From state *D*, animals cease showing clinical signs at rate *κ *but continue to be infectious – this is state *C *(carrier)

(2) The number of animals that are infectious but do not show clinical signs		
SELIRS		This does not occur in SELIRS; only animals showing clinical sign are infectious
SELDCRS	*C*	From state *C*, animals move to state *R *(recovered) at rate *α*, and hence are no longer infectious

For purposes of comparing the SELDCRS model with quantitative genetic models that ignore the transmission of infection (see below), the pathogen burden (*E*) in the population can be made independent of changes in the rate at which diseased animals apparently recover (*κ*) if any reductions in the number of diseased animals (*D*) are compensated by an increase in the number of carriers (*C*), *i.e*. the total time that an animal is infectious (1/*κ*+1/*α*) is kept constant. Properties of the SELDCRS model are derived in Appendix 1, with R', the reproductive rate of the disease, being defined as:

(13) $$R'=\frac{\phi \xi N}{\chi }(\frac{1}{\kappa }+\frac{1}{\alpha }).$$

and the equilibrium prevalence p*, being defined as:

(14) $$p*=\frac{\lambda \upsilon (1-\frac{1}{R'})}{\upsilon \lambda +\lambda \kappa +\upsilon \kappa +\lambda \nu \kappa /\alpha }.$$

Equation (14) can alternatively be written as:

(15) $$p*=\frac{\lambda \upsilon (1-\frac{1}{R'})}{\lambda +\upsilon +\upsilon \lambda (\frac{1}{\kappa }+\frac{1}{\alpha })}\frac{1}{\kappa }.$$

There are two differences between the equations defining equilibrium prevalence in the SELIRS and SELDCRS models (equations 8 and 15). First, if the assumption is invoked that the total infectious period is kept constant, then with decreasing 1/*κ *the term *R' *is constant in SELDCRS but will decrease with decreasing 1/*γ *in SELIRS. Secondly, under the same assumption of constant infectious period, inspection of equations 8 and 15 reveals that the SELDCRS model will lead to the lower equilibrium prevalence, given the same values for all other parameters. The equilibrium prevalence will be relatively insensitive to change in *R' *if it has a high value (*i.e*. not close to 1).

### Predicting responses to selection

Improvement of resistance to disease by genetic selection in its simplest form, *i.e*. disregarding information from relatives, consists of selection of those animals that do not show clinical signs at the time point at which the observations are made. The expected response to selection on such a binary trait depends on the heritability of resistance and the prevalence. This can be calculated assuming a threshold ('all or none') model with an underlying normally distributed liability with heritability *h*_L_^2^, which depends on the heritability of the binary trait (*h*_01_^2^) as:

*h*_L_^2 ^= *p*(1 - *p*)*z*^-2^*h*_01_^2^,

where *p *is the prevalence of the binary trait and *z *is the ordinate of the standardised normal distribution corresponding to *p *[13].

For the case where the number of healthy animals available for breeding exceeds the number required for selection, and again disregarding information from relatives, the response to selection is calculated as if the selected proportion is equal to 1-*p*, and on the underlying scale the response is *R*_L _= *ih*_L_^2 ^with *i *the selection intensity corresponding to 1-*p*. Because of reductions in prevalence, the response to selection is not linear but decreases with increasing values of 1-*p*.

In the context of the disease dynamics, selection for 'healthy' animals may be thought of as selection on one or more of the following:

a. The recovery rate *γ *or *κ *(*i.e*. infectious or diseased animals recover quicker, *e.g*. due to an acquired immune response)

b. The susceptibility of animals *ξ *(*i.e*. susceptible animals are more resistant to infection *per se*, requiring exposure to a greater number of infectious doses before becoming infected)

c. The rate of shedding of bacteria *φ *(*i.e*. infectious animals spread fewer bacteria) – this is comparable to selection for reduced egg counts with nematode infections

d. The rate at which immunity is lost *λ *(*i.e*. longer lasting immunity)

e. The rate at which latently infected animals become infectious *ν *(*i.e*. longer latency, as seen in scrapie).

Note that *ξ *and *φ *occur only as products in *R' *and p*, therefore their effects can be considered jointly in this model.

In the simplest analogy to the threshold model, one can think of the model parameters, particularly *γ *and *κ *(*i.e*. recovery rate) or *ξ *(susceptibility) as being analogous to the liability, with heritable between-animal variation. This being the case, we can then assume that the liability and one or more of these parameters have the same distribution and heritability, and responses to selection can be calibrated between the threshold model and the SELIRS or SELDCRS models. Note that because of (6) selection on *ξ *(or *φ*) is equivalent to selection on *R'*, given a constant *γ*.

The response to selection for a binary disease trait can now be estimated in two ways; based on the threshold model and on the SELIRS or SELDCRS model. The threshold model is fully described by the prevalence and heritability of resistance, but the epidemiological models require a greater number of parameters. While estimates for most are available, prediction of the response to selection also requires knowledge of variation in the parameter under selection. One way to approach this is to parameterise the threshold model and the epidemiological models so that (for example) they give the same predicted response to one round of selection, and then study the longer-term predicted selection responses from these models.

In the threshold model, the response to selection on the observed scale is equal to the change in prevalence p*_1_-p*_0_, with p*_1 _being the prevalence predicted in the threshold model after one round of selection. For the epidemiological model to predict the same response to selection we use the standard selection response equation *ih*^2^*σ*_x _= *p*_1 _*-*p*_0 _*, so that

(16) *σ*_x _= (*p*_1 _*-*p*_0 _*)/*ih*^2^,

with *σ*_x _being the implied phenotypic standard deviation of the trait under selection. This standard deviation can now be used for parameters in the SELIRS and SELDCRS models, in order to calibrate selection responses. Some properties of responses to selection for these models and their dependence on various parameters are derived in Appendix 1.

If selection is on 1/*R'*, or one of its animal-components other than *γ*, *i.e. ξ *or *φ*, and assuming that 1/*R' *is normally distributed, the phenotypic standard deviation *σ*_x _that leads to the same response to one round of selection in the SELIRS model as the threshold model (p*_1_-p*_0_) can be calculated (see Appendix 1) as

(17) $$\sigma_{x}=\frac{\left(\upsilon \lambda +\lambda \gamma +\upsilon \gamma )(p{*}_{0}-p{*}_{1}\right)}{\lambda \upsilon ih^{2}}.$$

### Application to footrot

To apply the SELIRS and SELDCRS models to footrot and predict likely effects of selection, parameters were chosen based on literature estimates of the length of time (in days) that each phase lasts, as shown in Table 3. Note that, by definition, the required rates are the inverse of the length of time.

**Table 3:** Published estimates of length of time (in days) of phases of the SELIRS model for footrot infection

*Phase (parameter)* Trait definition	Length (days)	Source
Latency (*ν*)		

Positive for *D. nodosus*	4	[16]
Positive for *D. nodosus*	5, 6	[17]
Signs of early footrot	8, 9	[16]
Typical signs of early footrot	7	[17]
Footrot observed in 13 out of 16 sheep	< 10	[18]
Footrot	10–14	[19]
*Bacterial survival *(*χ *= *μ *+ *ωN*)		

Bacterial survival	≤ 14	[20]
Bacterial survival to the extent they are still infectious	≤ 7 days	[20]
Bacterial survival	< a few days	[21]
Bacterial survival	≤ 4	[22]
Required resting period of pasture to avoid infection	7	[19]
*Duration of infection *(*γ*)		

Mean (may include re-infection)	190–208	[23]
Mean (may include re-infection)	21–77	[24]
With prompt treatment	1	
*Duration of immunity after recovery* (*λ*)		

Since start of treatment (and cured after 0–21 days)	26–31	[16]
Re-infection	< 63	[11]

To investigate the consequences of a range of values for *γ *and *R'*, the p*, *λ *and *ν *were set at 0.08, 0.0333 day^-1 ^and 0.1667 day^-1 ^respectively, and *γ *varied from 0.025, 0.1, 0.2 to 0.3 day^-1^so that corresponding values for *R' *were 1.18, 1.58, 2.91 and 18.08. Extreme values were investigated with *R' *= 20, *γ *= 0.025 day^-1 ^and *p*_0 _= 0.5.

To investigate the predicted response to selection in the various models and parameter combinations, the equilibrium prevalence of footrot was calculated over 20 rounds of selection on 1/*κ *and 1/*R' *in the SELDCRS and SELIRS models, using discrete generations and a heritability of 0.3, assuming a normal distribution for 1/*γ*, 1/*κ *and 1/*R' *with constant underlying variances and ignoring the Bulmer effect (whereby genetic variation is reduced as a consequence of selection). With the initial prevalence of footrot set at 0.08, the response to one round of selection using a threshold model was calculated to estimate *σ*_x _according to (16) and (17). Then (14) and (8) were used to estimate *R' *given the other parameters. Changes in the parameter under selection (1/*γ*, 1/*κ *or 1/*R'*) that would result from selection of a random sample of healthy animals were calculated for each generation and the new value inserted in (14) or (8) as appropriate to obtain the equilibrium prevalence in the next generation.

## Results

### Predicted progress in the threshold, SELDCRS and SELIRS models

**Figure 1:**
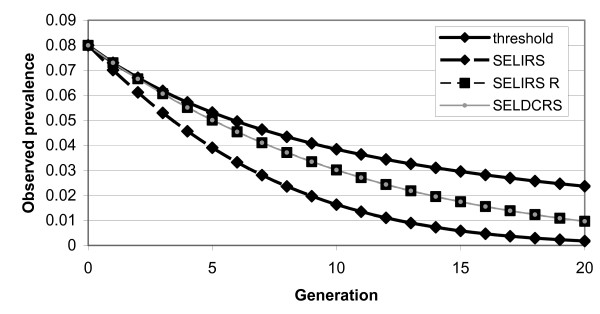
**Predicted response to selection for resistance to footrot depending on the model and the trait under selection**. The notional reproductive rate *R'*, or the recovery rate *γ *or *κ *is the trait under selection; initial values are *R' *= 2.91, *γ *= *κ *= 0.2, *λ *= 0.0333, *ν *= 0.1667, p* = 0.08 and *h*^2 ^= 0.3; the response is standardised to the same genetic response after one generation of selection, the SELIRS model with selection on *γ *shows an additional epidemiological effect.

Predicted responses to selection according to the SELDCRS model with selection on 1/*κ *(the time an animal is diseased and infectious) and the SELIRS model with selection on 1/*R' *are identical for values of *κ *= 0.2 and *R' *= 2.91 (Fig. 1). Both responses are larger than the threshold model prediction after the first round (when they were fixed at the same value). This is the result of differences in the relationship between the trait under selection and prevalence which is close to linear for SELIRS and SELDCRS but not for the threshold model. As expected, the SELIRS model predicts a significant additional response from selection on 1/*γ *compared to the SELDCRS model, in which the total infection period is held constant in this parameterisation. All graphs have a similar shape, showing diminishing returns at lower prevalences.

### Sensitivity of the predicted response to selection to *γ *and R'

**Figure 2:**
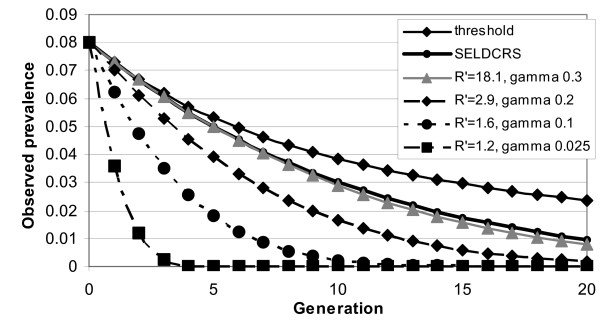
**Predicted response to selection for resistance to footrot depending on the model used, the notional reproductive rate *R' *and the initial recovery rate *γ***. Selection is on *γ*, with *λ *= 0.0333, *ν *= 0.1667, p* = 0.08 and *h*^2 ^= 0.3.

A value of *R' *of just greater than 1 leads to predicted prevalence of footrot quickly going to 0 (Fig. 2). The reason is that *R' *drops below 1, so that the infection is not expected to be maintained in the population. In contrast, very high values of *R' *(such as 18) do not lead to a noticeable additional predicted response. With typical values for *R' *for footrot of about 1.5 an additional response in prevalence of over 2% of animals can be expected after a few generations, but this difference decreases again in later generations.

**Figure 3:**
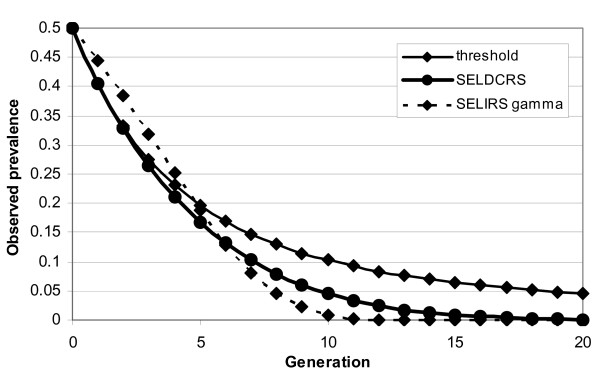
Predicted response to selection for resistance to footrot depending on the model and with selection on the recovery rate *γ*, initial values for the notional reproductive rate *R' *= 20 and *γ *= 0.025 and *λ *= 0.05, *ν *= 0.0625, p* = 0.5 and *h*^2 ^= 0.3.

For more extreme cases, with *R' *and p* large and *γ *small, a different picture emerges with the SELIRS model actually predicting a lower response to selection for larger *γ *in early generations than SELDCRS or selection on *R' *(Fig. 3). This is because the variation in 1/*γ *is relatively small and changes have little effect on *R'*.

### Sensitivity to *ν *and *λ*

Additional calculations (results not shown) have confirmed that the predicted response to selection on *γ *or *R' *does not depend on values of the rate at which latently-affected animals become infectious (*ν*) or the rate at which recovered animals lose immunity (*λ*), given *γ*, *R' *and p*.

## Discussion

A model was developed to predict the response to selection for resistance to an endemic bacterial disease, and this model was then applied to footrot in sheep. While the exact description of the epidemiological process requires a great number of parameters, many of which are poorly known, the prediction of relative responses to selection depends on only a few parameters. By using the threshold model to predict the response to one round of selection, and setting this as the standard, the only epidemiological parameters required are the rate at which animals recover (*γ*) and the reproductive rate (*R'*), alongside the heritability of resistance to the disease (or the parameter under selection).

It was shown that if the notional reproductive rate *R' *is under selection, changes in the prevalence are proportional to changes in 1/*R'*. If selection is for larger *γ *(*i.e*. quicker recovery) the SELIRS model predicts a response that consists of the direct effect of animals recovering more quickly, plus an additional component arising from the resulting lower pathogen burden.

It seems counterintuitive that there is no such additional 'epidemiological' component from selection on *R'*, especially since selection on *R' *may in fact be on the susceptibility to infection as denoted by *ξ*, and which is a component of *R'*. The expectation might be that if fewer animals get infected, there should also be a reduction in pathogens. Formula (7) shows that the equilibrium prevalence only depends on *ξ*, *i.e*. susceptibility, through *R'*. An explanation is that a decrease in susceptibility only means that it takes longer for animals to get infected when facing the same challenge (1/*ξ *days), but eventually they still become infected and shed the same number of bacteria. The advantage of a lower susceptibility, *i.e*. longer time until infection, is that it takes animals longer to complete the full SELIRS cycle, thereby reducing the number of animals that are diseased at any point in time prior to the equilibrium being reached.

Raadsma *et al*. [11] and Nieuwhof *et al*. [10] have estimated the heritability for resistance to footrot based on the genetic variation within a population, rather than the response to selection. Therefore, these estimates are independent of possible effects of reduced pathogen burden in selected populations.

Little information is available on the value of the recovery rate *γ*, and one reason is that prompt treatment of affected animals means it is not fully expressed (*i.e*. 1/*γ *is censored). The prevalence without treatment could then be considerably higher and *R' *larger. This scenario was investigated with an initial prevalence of 50%, and it was found that in early generations the expected additional effects are in fact negative, but the effect becomes positive later on. The reason for this negative effect, best understood by comparing (8) and (14) is that with increasing recovery rate in the SELDCRS model animals spend increasing times in the *C *phase, *i.e*. they are no longer considered diseased but continue to spread bacteria. This slows down the whole cycle, with there being fewer susceptible animals compared to the SELIRS model, where recovered animals move directly to the phase of immunity. For our application to footrot this effect may be considered an artefact of the model, occurring only under extreme assumptions, rather than of biological importance, but it may be relevant for other diseases. Previously, MacKenzie and Bishop [14] have shown that in the SIR model applied to viral diseases, if *R*_0 _is high then it may take many generations of selection before the expected number of animals infected during an epidemic is expected to decrease. This, also, is a scenario in which the epidemic model predicts a slower response to selection than the quantitative genetic model.

Knowledge of the traits that show genetic variation is clearly important. While the current results suggest that this can be done by comparing the variation within a population with the response to selection, in practice this will be very difficult because it would require an unselected control or population with the same environmental conditions, but not affected by decreased shedding of bacteria by the selected animals. An alternative would be to estimate parameters directly from the length of time various epidemiological stages last, in selected and unselected populations following (deliberate) infection, comparable to the figures in Table 3.

In the absence of any estimates for the genetic variance of resistance to footrot, this study used the threshold model to standardise response to selection. Following the concept of an underlying normally distributed trait in the threshold model, normal distribution were assumed for 1/*R' *and 1/*γ*. The inverse of the recovery rate 1/*γ *is a length of time, and it seems plausible that it has a positive skewness, with no negative values, and some animals taking extremely long to recover. A positive skewness looks likely for *R' *as well, especially for scenarios where mean *R' *is in the critical range just above 1, with some animals potentially being extremely infectious. Positively skewed distributions for the inverse 1/*γ *and 1/*R' *would for instance occur if *γ *and *R' *were normally distributed. Under these scenarios, relative responses to selection can be recalculated with appropriately altered selection intensities. However, it should be remembered that a normally distributed liability in the threshold model is also an assumption that can be challenged.

It was shown that, under the prevailing assumptions, the reduction in prevalence at a given *R' *does not depend on the rate of loss of immunity *λ *and the rate of conversion of latently infected animals to the infectious state *ν*. This does not mean that these parameters are not important for the potential genetic progress; it implies that once the response to one round of selection is known it is possible to predict further response without knowing the values of *λ *and *ν*.

In the current study, a constant environment and a homogeneous population have been assumed. In practice, environmental conditions will vary and this may affect survival of bacteria in the environment or the animals' phenotypes, while there may also be different classes of animals, *e.g*. adults and offspring with different phenotypes with regard to the disease. All these variations can be investigated based on the SELIRS equations, but may require running of a dynamic algorithm that calculates daily prevalence, rather than relying on equilibria. In rapidly changing environments the time to reach equilibrium will become an important factor, with potentially no equilibrium being attained by the time prevalence is measured or selection decisions are made. In a stable environment, changes in parameters as a result of selection will lead to only small changes in the expected equilibria so that a new equilibrium can quickly be established.

Selection in this study was based on own performance and one observation per animal with disease resistance as the only breeding goal. In practice, information from relatives and repeated measurements will increase the response to selection. On the one hand, assuming that resistance to footrot is not genetically correlated to any other traits under selection, selection on an index of traits will decrease the expected response to selection for resistance. On the other hand, disease information on relatives will greatly improve the potential selection response rates for improved resistance. While all these considerations affect the magnitude of the response to selection, essentially by changing the 'h^2^' term in the response equations, they do not alter the nature of the epidemiological effects of selection. Therefore, simple extrapolation is appropriate.

The models developed in this study are used to consider an endemic bacterial disease with bacteria being transmitted through the environment, where they can only survive for a limited period of time. The models can be applied to a variety of diseases and host species, where these conditions apply. The general trend of results is in fact similar to that seen for a different disease, ruminant gastro-intestinal parasitism, as shown by Bishop and Stear [15]. One difference is that these authors had better estimates of some traits, especially the rate at which animals spread infection, as this is captured in the faecal egg count trait.

Based on the current study it can be expected that selection for resistance to footrot in sheep will be more considerably effective, especially in the medium term, than purely genetic models predict. There are, however, many other important issues to consider in a practical breeding programme, such as obtaining consistent disease scores across a population of sufficient size and the simultaneous selection for other traits, which may be correlated, on a phenotypic or genetic level, with resistance to footrot.

In summary, this paper presents a novel epidemic model, applied to footrot in an attempt to explore likely responses to selection. A key parameter for the model, and also from a biological perspective, is the recovery rate. Given the long time that it takes animals to recover from the disease without human intervention, low values for the rate of recovery (*γ*) seem likely. If this is indeed the trait under selection when selecting for increased resistance, then the response to selection in terms of observed prevalence, including effects of reduced pathogen burden, could in the medium term be double that predicted by purely genetic models.

## Appendix 1

### Derivation of R' for the SELIRS model

Assuming that *N *is large, so that *S *is approximately equal to *N*, in the SEIR model an infected animal sheds *φ *infectious doses over 1/*γ *days, these doses survive for 1/*χ *days infecting *N*/*ξ *daily so that *R' *= *φξN*/*γχ*. A more formal derivation is given in [8].

The extra *L *step in the SELIRS model does not affect this, as all latently infected animals will (sooner or later depending on *υ*) become diseased. For most parameter values, the loss of immunity (*R *animals reverting to *S*) does not affect the number of secondary infections, but extreme parameter values (long-lived environmental contamination combined with a short period of immunity) may lead to more secondary infections.

### Derivation of numbers of animals in various categories at the equilibrium in the SELIRS model

At the equilibrium (denoted by *) all derivatives, d*I*/d*t *etc are equal to 0, so that from (4) it follows that:

(A1) γI* = νL* = *ν(N*-S*-I*-R*).

From (5) R* = γI*/*λ*, and combining (3) and (4) gives S* = γI*/ξE*, while from (2) E* = φI*/*χ*, so that S* = *γχ*/*ξφ*. Substituting into (A1) then gives:

γI* = *ν(N*-*γχ*/*ξφ*-I*-γI*/λ),

Rearranging and solving for I* yields:

$$I*=\frac{\lambda \upsilon (N-\frac{\gamma \chi }{\xi \phi })}{\lambda \upsilon +\lambda \gamma +\upsilon \gamma }.$$

### Summary parameters in the SELDCRS model

Following Appendix A, *R' *for the SELDCRS model can be calculated from the product of number of infectious doses produced at rate *φ *by an infectious animal over the duration of the infection (1/*κ*+1/*α*), the time these infections survive in the environment (1/*χ*) and the number of animals that will be infected at a rate of *ξ N*, so that:

$$R'=\frac{\phi \xi N(1/\kappa +1/\alpha )}{\chi }.$$

As with the SELIRS model in Appendix B, the equilibrium prevalence p* can be calculated by setting all derivatives to 0. Combining (3) and (10) then gives S* = κD*/ξE*, and from (9) and (11) it follows that

$$E*=\frac{\phi (D*+\frac{\kappa D*}{\alpha })}{\chi }$$

so that:

$$S*=\frac{\chi }{\xi \phi (\frac{1}{\kappa }+\frac{1}{\alpha })}.$$

From (10), (11) and (12), L* = κD*/*ν*, C* = κD*/*α *and R* = κD*/*λ*. Substituting these values in *L+D+C+R *= *N-S *and rearranging yields:

$$D*=\frac{\lambda \upsilon \alpha N(1-\frac{\chi }{\xi \phi (\frac{1}{\kappa }+\frac{1}{\alpha })})}{\upsilon \lambda \alpha +\lambda \kappa \alpha +\upsilon \kappa \alpha +\lambda \nu \kappa },$$

Therefore, from (14) the equilibrium prevalence is

$$p*=\frac{\lambda \upsilon (1-\frac{1}{R'})}{\upsilon \lambda +\lambda \kappa +\upsilon \kappa +\lambda \nu \kappa /\alpha }.$$

### Derivation of the predicted response to selection on *γ *or *κ *and demonstration that the rate of response in p* depends on *λ *and *ν*, given *R*' and p* in the SELDCRS and SELIRS models

#### SELDCRS model

From (15) $p*=\frac{1-\frac{1}{R'}}{K\kappa +\kappa (\frac{1}{\lambda }+\frac{1}{\nu })}$, with $K=\frac{1}{\kappa }+\frac{1}{\alpha }$ (K set to be constant).

Therefore $\frac{1}{p*}=\frac{\kappa (K+\frac{1}{\lambda }+\frac{1}{\nu })}{1-\frac{1}{R'}}$.

Now consider the derivative d(1/p*)/d*κ*. Solving this equation we obtain:

$$-\frac{\text{1}}{p{*}^{2}}\text{d}p*/\text{d}\kappa =\frac{K+\frac{1}{\lambda }+\frac{1}{\nu }}{1-\frac{1}{R'}}.$$

Therefore:

$$\text{d}p*/\text{d}\kappa =-\frac{(K+\frac{1}{\lambda }+\frac{1}{\nu })p{*}^{2}}{1-\frac{1}{R'}},$$

Thus, in the SLEDCRS model the predicted response in p* to changes in *κ *is a linear function of (1/*λ *+ 1/*ν*), and it also depends on *R' *and p* but under the model assumptions it is independent of *κ*.

#### SELIRS model

Define a new parameter *Q' *so that $Q'=\frac{\phi \xi N}{\chi }$ and *Q' *is independent of *γ*. Since *Q' *= *R'γ *we have:

(D1) $$p*=\frac{1-\frac{\gamma }{Q'}}{1+\gamma (\frac{1}{\lambda }+\frac{1}{\nu })}.$$

Equation (D1) can be rearranged as

$$\frac{1}{p*}(1-\frac{\gamma }{Q'})=1+\gamma (\frac{1}{\lambda }+\frac{1}{\nu })$$

Solving the derivative d(1/p*)/d*γ *we obtain:

$$\frac{-1}{p{*}^{2}}(1-\frac{\gamma }{Q'})\text{d}p/\text{d}\gamma -\frac{1}{p*}\frac{1}{Q'}=\frac{1}{\lambda }+\frac{1}{\nu }$$

Rearranging terms yields:

(D2) $$\text{d}p*/\text{d}\gamma =\frac{-p*}{Q'-\gamma }-\frac{Q'(\frac{1}{\lambda }+\frac{1}{\nu })p{*}^{2}}{Q'-\gamma }.$$

There are two important differences from the derivative for p* in the SELDCRS model. Firstly, there is the extra element $\frac{-p*}{Q'-\gamma }$, which is more important at low values of *Q*' (*i.e*. low values of *γR'*). Also, the second part of the equation includes *γ*, so that higher values of *γ *lead to higher rates of change.

### Calculation of a term for the implied phenotypic standard deviation for 1/R' in the SELIRS model

The predicted genetic response to one round of selection is:

*ih*^2^*σ*_x _= 1/*R'*_1_-1/*R'*_0_.

From (8) the resulting difference in prevalence, i.e. p*_1_-p*_0_, is:

p1−p0=λυ(1−1R1')υλ+λγ+υγ−λυ(1−1R0')υλ+λγ+υγ=λυ(1R0'−1R1')υλ+λγ+υγ=λυih2σxυλ+λγ+υγ

Rearranging gives:

$$\sigma_{x}=\frac{\left(\upsilon \lambda +\lambda \gamma +\upsilon \gamma )(p_{0}-p_{1}\right)}{\lambda \upsilon ih^{2}}.$$

## Competing interests

The authors declare that they have no competing interests.

## Authors' contributions

GJN developed the model, derived the mathematical equations, performed all calculations and drafted the manuscript. JC advised on footrot biology and epidemiology, assisted in the writing of the manuscript and led the larger research project on footrot, of which this is a component. SCB conceived the model and assisted in the writing of the manuscript. All authors read and approved the final manuscript.

## References

[B1] Bennett RM, IJpelaar ACE (2003). Economic assessment of livestock diseases in Great Britain. Final Report to the Department for Environment, Food and Rural Affairs.

[B2] Archibald AL, Bishop SC (2006). S11: State-of-Science Review – Host Genetics and Engineering: the genetics of host responses to infectious diseases in farmed animals. Foresight project 'Infectious Diseases: preparing for the future'.

[B3] Axford RFE, Bishop SC, Nicholas FW, Owen JB (2000). Breeding for disease resistance in farm animals.

[B4] Bishop SC, Stear MJ (2003). Modelling host genetics and resistance to infectious diseases: understanding and controlling infections. Vet Parasitol.

[B5] Anderson RM, May RM (1991). Infectious diseases of humans, dynamics and control.

[B6] Bishop SC, MacKenzie KM (2003). Genetic management strategies for controlling infectious diseases in livestock populations. Genet Sel Evol.

[B7] Nath M, Woolliams JA, Bishop SC (2004). Identifying critical parameters in the dynamics and control of microparasite infection using a stochastic epidemiological model. J Anim Sci.

[B8] Bishop SC, Woolliams JA, Wallis IP (2006). Developing genetic epidemiological models for bacterial infections with environmental contamination. 8th World Congress on Genetics Applied to Livestock Production: 13–18 August 2006; Belo Horizonte, Brazil.

[B9] Nieuwhof GJ, Bishop SC (2005). Costs of the major endemic diseases of sheep in Great Britain and the potential benefits of reduction in disease impact. Anim Sci.

[B10] Nieuwhof GJ, Conington J, Bûnger L, Haresign W, Bishop SC (2008). Genetic and phenotypic aspects of foot lesion scores in sheep of different breeds and ages. Animal.

[B11] Raadsma HW, Egerton JR, Wood D, Kristo C, Nicholas FW (1994). Disease resistance in Merino sheep. III. Genetic variation in resistance to footrot following challenge and subsequent vaccination with an homologous rDNA pilus vaccine under both induced and natural conditions. J Anim Breed Genet.

[B12] Skerman TM, Johnson DL, Kane DW, Clarke JN (1988). Clinical footscald and footrot in a New Zealand Romney flock: phenotypic and genetic parameters. Aust J Agric Res.

[B13] Robertson A, Lerner IM (1949). The heritability of all-or-none traits: viability of poultry. Genetics.

[B14] MacKenzie KM, Bishop SC (1999). A discrete-time epidemiological model to quantify selection for disease resistance. Anim Sci.

[B15] Bishop SC, Stear MJ (1999). Genetic and epidemiological relationships between productivity and disease resistance: gastro-intestinal parasite infection in growing lambs. Anim Sci.

[B16] Egerton JR, Roberts DS, Parsonson IM (1969). The aetiology and pathogenesis of ovine foot-rot. I. A histological study of the bacterial invasion. J Comp Path.

[B17] Roberts DS, Egerton JR (1969). The aetiology and pathogenesis of ovine foot-rot. II. The pathogenic association of *Fusiformis nodosus *and *F. necrophorus*. J Comp Pathol.

[B18] Egerton JR, Roberts DS (1971). Vaccination against ovine foot-rot. J Comp Path.

[B19] Egerton JR, Martin WB, Aitken ID (2000). Foot-rot and other conditions. Diseases of sheep.

[B20] Beveridge WIB (1941). Foot-rot in sheep; a transmissible disease due to infection with *Fusiformis nodosus *(n.sp.): Studies on its cause, epidemiology and control. CSIRO Aust Bull.

[B21] Laing EA, Egerton JT (1981). Some aspects of *B. nodosus *infection in cattle. Ovine footrot Report of a Workshop.

[B22] West DM, Bruère AN, Ridler AL (2002). The sheep: Health, disease & production.

[B23] Abbott KA, Egerton JR (2003). Effect of climatic region on the clinical expression of footrot of lesser clinical severity (intermediate footrot) in sheep. Aust Vet J.

[B24] Egerton JR, Ribeiro LA, Kieran PJ, Thorley CM (1983). Onset and remission of ovine footrot. Aust Vet J.

